# Protective Effects of Selenium Nanoparticles against Bisphenol A-Induced Toxicity in Porcine Intestinal Epithelial Cells

**DOI:** 10.3390/ijms24087242

**Published:** 2023-04-14

**Authors:** Zaozao Pan, Jiaqiang Huang, Ting Hu, Yonghong Zhang, Lingyu Zhang, Jiaxi Zhang, Defeng Cui, Lu Li, Jing Wang, Qiong Wu

**Affiliations:** 1 Animal Science and Technology College, Beijing University of Agriculture, Beijing 100096, China; 2 Beijing Advanced Innovation Center for Food Nutrition and Human Health, Department of Nutrition and Health, China Agricultural University, Beijing 100193, China; 3 Institute of Animal Husbandry and Veterinary Medicine, Beijing Academy of Agriculture and Forestry Sciences, Beijing 100097, China

**Keywords:** Bisphenol A, selenium nanoparticles, tight junction, oxidative stress, apoptosis, endoplasmic reticulum stress

## Abstract

Bisphenol A (BPA) is widely used to harden plastics and polycarbonates and causes serious toxic effects in multiple organs, including the intestines. Selenium, as an essential nutrient element for humans and animals, exhibits a predominant effect in various physiological processes. Selenium nanoparticles have attracted more and more attention due to their outstanding biological activity and biosafety. We prepared chitosan-coated selenium nanoparticles (SeNPs) and further compared the protective effects, and investigated the underlying mechanism of SeNPs and inorganic selenium (Na_2_SeO_3_) on BPA-induced toxicity in porcine intestinal epithelial cells (IPEC-J2). The particle size, zeta potential, and microstructure of SeNPs were detected by using a nano-selenium particle size meter and a transmission electron microscope. IPEC-J2 cells were exposed to BPA alone or simultaneously exposed to BPA and SeNPs or Na_2_SeO_3_. The CCK8 assay was performed to screen the optimal concentration of BPA exposure and the optimal concentration of SeNPs and Na_2_SeO_3_ treatment. The apoptosis rate was detected by flow cytometry. Real-time PCR and Western blot methods were used to analyze the mRNA and protein expression of factors related to tight junctions, apoptosis, inflammatory responses and endoplasmic reticulum stress. Increased death and morphological damage were observed after BPA exposure, and these increases were attenuated by SeNPs and Na_2_SeO_3_ treatment. BPA exposure disturbed the tight junction function involved with decreased expression of tight junction protein Zonula occludens 1 (ZO-1), occludin, and claudin-1 proteins. Proinflammatory response mediated by the transcription factor nuclear factor-k-gene binding (NF-κB), such as elevated levels of *interleukin*-*1β(IL*-*1β)*, *interleukin*-*6 (IL*-*6)*, *interferon*-*γ (IFN*-*γ)*, *interleukin*-*17 (IL*-*17)*, and tumor necrosis *factor*-*α* (*TNF*-*α)* expression was induced at 6 and 24 h after BPA exposure. BPA exposure also disturbed the oxidant/antioxidant status and led to oxidative stress. IPEC-J2 cell apoptosis was induced by BPA exposure, as indicated by increased BCL-2-associated X protein (Bax), caspase 3, caspase 8, and caspase 9 expression and decreased B-cell lymphoma-2 (Bcl-2) and Bcl-xl expression. BPA exposure activated the endoplasmic reticulum stress (ERS) mediated by the receptor protein kinase receptor-like endoplasmic reticulum kinase (PERK), Inositol requiring enzyme 1 (IRE1α), and activating transcription factor 6 (ATF6). We found that treatment with SeNPs and Na_2_SeO_3_ can alleviate the intestinal damage caused by BPA. SeNPs were superior to Na_2_SeO_3_ and counteracted BPA-induced tight junction function injury, proinflammatory response, oxidative stress, apoptosis, and ERS stress. Our findings suggest that SeNPs protect intestinal epithelial cells from BPA-induced damage, partly through inhibiting ER stress activation and subsequently attenuating proinflammatory responses and oxidative stress and suppressing apoptosis, thus enhancing the intestinal epithelial barrier function. Our data indicate that selenium nanoparticles may represent an effective and reliable tool for preventing BPA toxicity in animals and humans.

## 1. Introduction

The intestinal tract is the organ with the largest surface area in direct contact with the external environment of the animal body, which has critical biological functions such as digestion, absorption, metabolism, and immunity [1]. The intestinal barrier mainly consists of four aspects: intestinal commensal microorganisms, chemical barrier, physical barrier, and immune barrier [2]. Among these, intestinal barrier integrity largely relies on the intestinal epithelial cells (IECs). The IEC is the first cell boundary between the luminal environment and the body against outside hostile stimuli. The choice of the IEC’s unique permeability can guarantee the body’s absorption of nutrients and effectively suppress pathogenic microorganisms and harmful substances through the barrier to enter the blood to maintain the body’s normal physiological function [3]. The IEC barrier’s permeability is regulated by the junction complex formed between adjacent intestinal epithelial cells [4]. The intercellular junction complex is mainly composed of tight junctions, which mainly include claudins, occludin, and ZO families [5]. Maintaining the normal expression and distribution of tight junction proteins is essential for intestinal barrier function.

BPA is one of the most widely used industrial compounds in the world, mainly in the production of plastic containers, toys, tableware, medical devices, and polycarbonate bottles. As an endocrine-disrupting chemical, BPA has estrogen-like and anti-androgen properties, causing significant damage to human tissues and organs, such as the reproductive system, immune system, and neuroendocrine system [6]. Humans and animals are exposed to BPA mainly through dietary, transdermal, and inhalation, in which dietary is considered the main route [7]. Studies have demonstrated that after BPA enters the human body, it first destroys the intestinal epithelial barrier functions, intestinal immune systems, and reproductive systems, and thus leads to the occurrence of various metabolic diseases [8]. Mice dietary intake of BPA first destroys the morphological structure of intestinal epithelial cells by inhibiting the expression of tight junction proteins and increasing intestinal permeability [9]. Similarly, the oral administration of BPA can affect the intestinal barrier functions and then accumulate in the intestine, liver, and gonads of pigs, leading to reproductive toxicity [10]. Further studies also indicated that dietary exposure to BPA destroys the gastrointestinal mucosal layer of human colon cancer cells (HCT116), leading to increased apoptosis and slower progression associated with intestinal epithelial cell proliferation [11]. BPA treatment increases intestinal permeability and disrupts the barrier function by increasing the chemical marker content and tight junction expression in intestinal tissues and blood circulation [12]. It remains unclear how BPA damages the intestinal epithelial barrier functions.

The endoplasmic reticulum (ER) is composed of branched tubules and flat reticular capsules that are present in all eukaryotic cells and extend into the cytoplasm to surround the nuclear membrane. The endoplasmic reticulum can be divided into two types: the rough endoplasmic reticulum full of ribosomes and the smooth endoplasmic reticulum rich in lipid synthetase. The rough endoplasmic reticulum is mainly involved in the biosynthesis, folding, processing and modification of soluble and membrane proteins. The rough endoplasmic reticulum is of paramount importance for adaptive responses to biotic stresses due to increased unfolded or misfolded proteins. ERS is well known as the accumulation of unfolded proteins in ER lumen caused by the increased protein synthesis or multiple conditions, and ER responds to ER stress by activating the unfolded protein response (UPR), an adaptive intracellular signal to cope with ER stress and help to sustain cell survival and normal functions [13]. In response to ER stress, ER-resident chaperone binding immunoglobulin protein (Bip) dissociates from the luminal domains of the three protein sensors, leading to the activation of proteins PERK, IRE1, and ATF6 to restore intracellular homeostasis [14,15]. Notably, ER stress in intestinal epithelium evokes a range of adverse cellular responses, such as oxidative stress, inflammatory responses, and apoptosis, which are all involved in the pathogenesis of intestinal barrier dysfunction [16,17,18]. Dietary intake of BPA results in an abnormal increase in the amount of unfolded and misfolded proteins in the ER and then induces ER stress [19]. Studies have shown that BPA can reveal persistent ER stress by activating ATF6 and IRE1 pathways in mammals [20]. Moreover, BPA activates ER stress and stimulates oxidative stress, thereby promoting cell apoptosis and damaging intestinal barrier functions [21].

As an essential nutrient element for humans and animals, selenium has important biological functions for human health, such as anti-cancer, anti-inflammatory, and anti-oxidation. In recent years, selenium has been applied in the form of inorganic selenium, organic selenium, and nano-selenium. Studies demonstrated that the use of inorganic sodium selenite ameliorates BPA toxicity in the liver, testis, and lungs of mice [22]. However, because inorganic selenium is easy to combine with vitamins and has poor stability, low bioavailability, and high biological toxicity, it is difficult to control the safe use dose in the actual use process [23]. In recent years, selenium nanoparticles, as a new type of elemental selenium, have attracted much attention. Compared with inorganic selenium and organic selenium, selenium nanoparticles have higher bioavailability, stronger biological activity, lower toxicity, and better compatibility as therapeutic drug carriers, and are easy to synthesize and store [24,25]. Previous studies have shown that the intestinal villus circumference and height of fish fed with a nano-selenium diet were greater than those of fish fed with sodium selenite, suggesting that nano-selenium can be more effective in maintaining intestinal integrity [26]. Recent studies have shown that, compared with inorganic selenium and organic selenium, selenium nanoparticles have obvious biological beneficial effects on the alleviation of cadmium-induced inflammation via NF-κB/IκB pathway in the heart [27]. Nano-selenium is superior to inorganic selenium and organic selenium in attenuating the cardiotoxic effects of cadmium by activating the aryl hydrocarbon receptor pathway [28]. Studies also have shown that selenium nanoparticles attenuate BPA-induced testicular toxicity by inhibiting oxidative stress in male rats [29]. The molecular mechanism underlying the protective effects of selenium nanoparticles against BPA-induced toxicity needs to be further explored.

In the present study, we established the model of BPA-stimulated porcine IPEC-J2 cells and further analyzed the effects of prepared SeNPs on ER stress and the downstream apoptosis, oxidative stress, and inflammatory pathways.

## 2. Results

### 2.1. Preparation and Characterization of SeNPs

**Figure 1:**
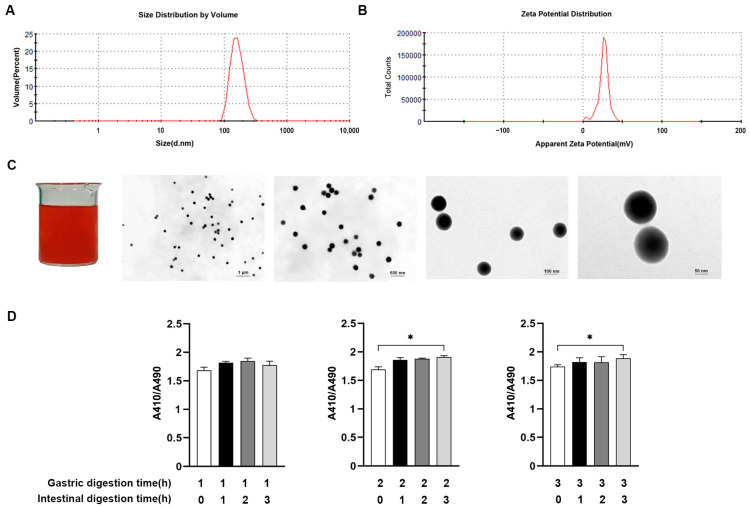
Determination of physicochemical properties and stability of SeNPs. The size distribution (**A**) and zeta potential (**B**) of prepared SeNPs were measured. Schematic diagram of macroscopic and microscopic morphology of SeNPs (**C**). Changes in particle size of SeNPs were measured after digestion in vitro (**D**). Data are presented as the means ± SEM of three independent experiments. * *p* < 0.05.

The size distribution, zeta potential, microstructure, and stability of particles of SeNPs are presented in Figure 1. SeNPs were prepared by in-situ reduction of selenite and vitamin C using chitosan as the carrier material, and the concentration of SeNPs was 76.424 mg/g. The characteristic peak of the absorption maximum for SeNPs was 149.7 nm, indicating the small particle size of SeNPs (Figure 1A). The zeta potential of SeNPs ranged from −45.8 to 31.4 mV, and a signature peak appeared at 27.7 mV (Figure 1B). The TEM images showed that the SeNPs were distributed as well-dispersed spherical particles (Figure 1C). The presence of pepsin and trypsin in the gastroenteric fluid in the digestive system affects the structure and particle size of SeNPs. The stability of SeNPs was measured after gastric and intestinal digestion. The absorption values of SeNPs did not change after 1–3 h of gastric digestion and 1–2 h of intestinal digestion but increased after 2–3 h of gastric digestion and 3 h of intestinal digestion (Figure 1D).

### 2.2. Establishment of the Model of BPA-Exposed and Na_2_SeO_3_ or SeNPs-Treated IPECs

**Figure 2:**
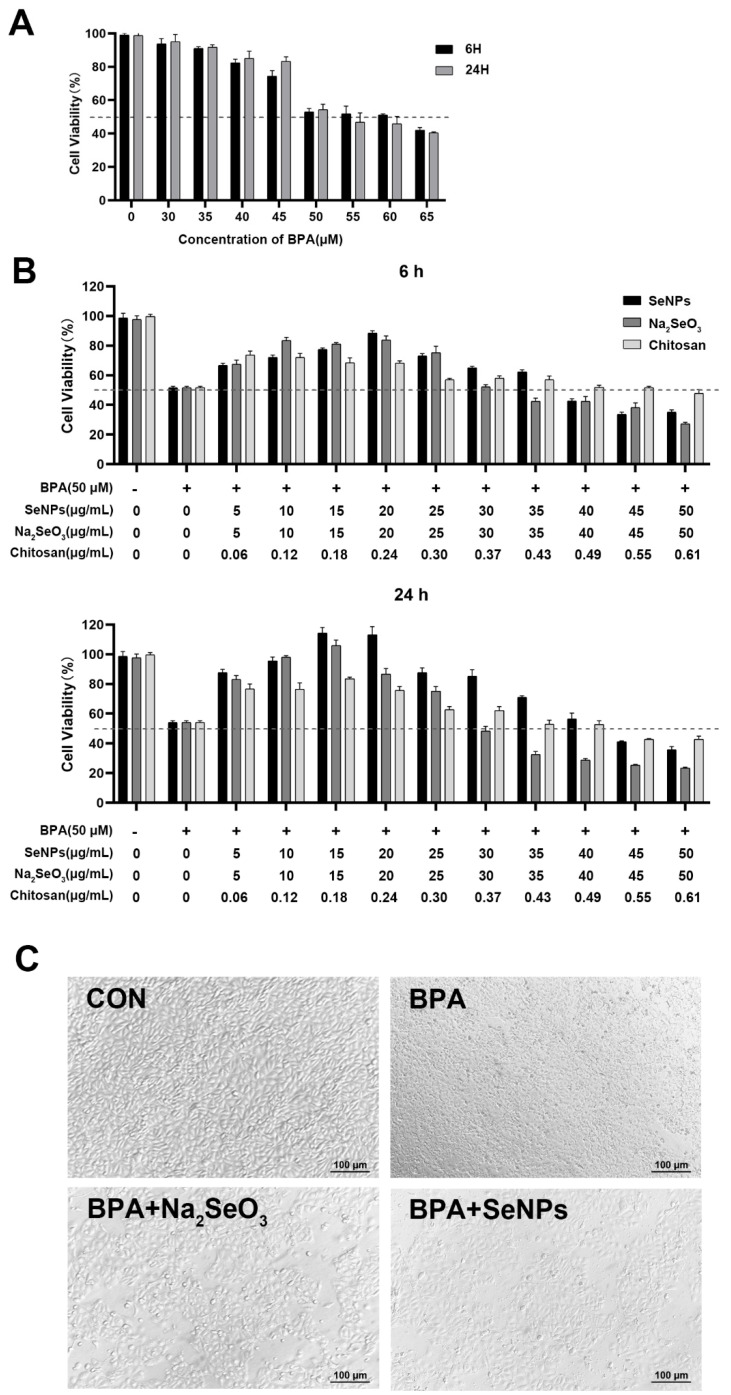
Effects of Na_2_SeO_3_ and SeNPs on viability and morphology of IPEC-J2 after BPA exposure. The dashed line represented the standard line of 50% cell viability. The optimal concentration of BPA was determined by measuring the cell viability after BPA exposure (**A**). The optimal concentration of Na_2_SeO_3_ or SeNPs was determined by measuring the cell viability when cells were treated with Na_2_SeO_3_ or SeNPs, followed by BPA exposure (**B**). The morphological changes of IPEC-J2 were observed when cells were treated with 15 μg/mL Na_2_SeO_3_ or SeNPs followed by 50 μM of BPA exposure for 24 h (**C**). Data are presented as the means ± SEM of three independent experiments.

The viability and morphological characteristics of IPECs were measured and observed to determine the optimal exposure dose of BPA and treatment dose of Na_2_SeO_3_ or SeNPs. Cell viability decreased when exposed to the increased BPA concentration, and cell viability decreased to about 60% at a BPA concentration of 50 μM, whether 6 or 24 h after BPA exposure (Figure 2A). Thus, we choose 50 μM as the BPA exposure concentration to allow for cell damage without disruption of the cell monolayer in subsequent experiments. After adding 5–50 μg/mL of SeNPs or Na_2_SeO_3_, a low dose of SeNPs or Na_2_SeO_3_ decreased the cell death induced by 50 μM of BPA, but a high dose of SeNPs or Na_2_SeO_3_ led to cell death. SeNPs or Na_2_SeO_3_ at the concentration of 15 μg/mL exhibited the best inhibitory effect on BPA-induced cell damage (Figure 2B). Under the optical microscope, at 24 h, untreated control cells appeared normal, and the cell monolayer was intact. At 24 h after BPA exposure, cells were broken, and the boundary was blurred. However, the addition of 15 μg/mL SeNPs or Na_2_SeO_3_ attenuated the degree of disruption of the cell monolayer induced by BPA. Compared with cells in the Na_2_SeO_3_ + BPA group, the number of vacuolar dead cells was less in the SeNPs + BPA group (Figure 2C).

### 2.3. SeNPs Maintained the TJ Expression in BPA-Exposed IPECs

**Figure 3:**
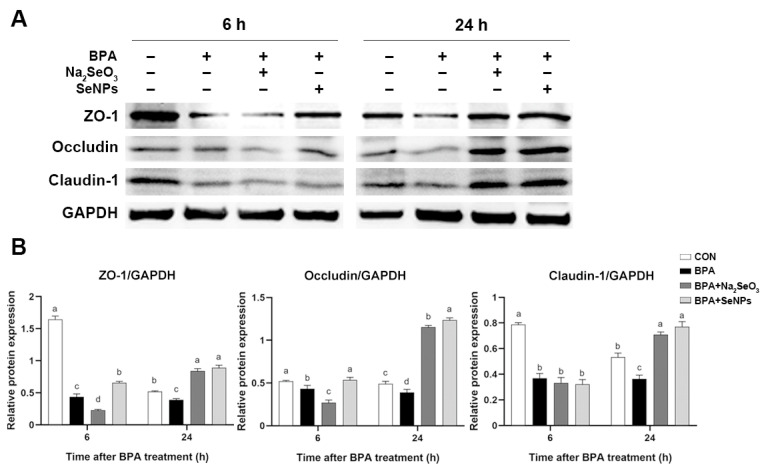
The effects of Na_2_SeO_3_ and SeNPs treatment on the TJ expression of IPEC-J2 cells after BPA exposure. Representative panels showing expression of ZO-1, Occludin, and Claudin-1 proteins by Na_2_SeO_3_ or SeNPs treatment followed by BPA exposure at 6 and 24 h (**A**). Expression of GAPDH was measured as an internal control. Results are presented as the ratio of ZO-1, Occludin, and Claudin-1 band intensity to that of GAPDH (**B**). Data are presented as the means ± SEM of three independent experiments. ^a,b,c,d^ Mean values within a row with different superscript letters were significantly different (*p* < 0.05).

To investigate the effect of SeNPs on intestinal integrity in BPA-exposed cells, the expression of tight junction proteins was determined. Compared with untreated control IPECs, the expression of ZO-1 and occludin proteins was decreased at 6 and 24 h after BPA exposure in cells only exposed to BPA (Figure 3A,B). Compared with cells only exposed to BPA, the expression of ZO-1 and occludin proteins was decreased at 6 h in the Na_2_SeO_3_ + BPA group but increased in the SeNPs + BPA group. Compared with untreated control IPECs, BPA exposure led to the decreased expression of ZO-1 and occludin proteins at 24 h, whereas Na_2_SeO_3_ or SeNPs treatment enhanced the ZO-1 and occludin proteins expression. BPA exposure led to decreased expression of claudin-1 protein at 6 h, regardless of with or without SeNPs or Na_2_SeO_3_ treatment. Compared with untreated control IPECs, BPA exposure led to the decreased expression of claudin-1 protein at 24 h, whereas Na_2_SeO_3_ or SeNPs treatment enhanced the claudin-1 protein expression.

### 2.4. SeNPs Attenuated the BPA-Induced IPEC Apoptosis

**Figure 4:**
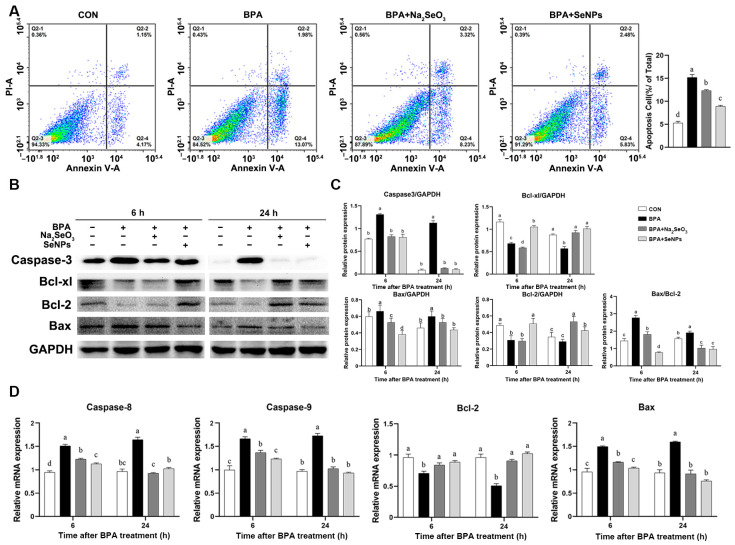
Effects of Na_2_SeO_3_ and SeNPs treatment on apoptosis of IPEC-J2 cells induced by BPA. Cell apoptosis was determined by flow cytometric analysis and analyzed (**A**). Representative panels showing expression of caspase 3, Bcl-xl, Bax, and Bcl-2 proteins by Western blotting at 6 and 24 h after BPA challenge (**B**). Expression of GAPDH was measured as an internal control. Results are presented as the ratio of caspase 3, Bcl-xl, Bax, and Bcl-2 protein band intensities to that of GAPDH, and the cellular protein Bcl-2 was used as an internal control for Bax (**C**). The relative expression of mRNAs for the *caspase-8*, *caspase-9*, *Bcl-2*, and *Bax* genes was analyzed by quantitative real-time PCR (**D**). Data are presented as the means ± SEM of three independent experiments. ^a,b,c,d^ Mean values within a row with different superscript letters were significantly different (*p* < 0.05).

According to the flow cytometry results, IPECs exposed to BPA alone had a higher percentage of apoptosis at 24 h compared with untreated control cells (Figure 4A). Treatment with SeNPs or Na_2_SeO_3_ resulted in a decrease in the percentage of apoptosis during BPA exposure. Cells in the SeNPs + BPA group had a lower percentage of apoptosis than cells in the Na_2_SeO_3_ + BPA group. Western blot results showed that BPA exposure increased the expression of pro-apoptotic protein caspase-3 and Bax at 6 and 24 h compared with untreated control cells, whereas treatment with SeNPs or Na_2_SeO_3_ attenuated this increase (Figure 4B,C). The BPA-induced down-regulation of the expression of anti-apoptotic protein Bcl-2 and Bcl-xl was observed, and this down-regulation was attenuated by SeNPs treatment at 6 and 24 h but not by Na_2_SeO_3_ treatment at 6 h. Consistently, treatment with SeNPs and Na_2_SeO_3_ reversed the increase in the mRNA expression of *caspase*-*8*, *caspase*-*9,* and *Bax* and the decrease in the mRNA expression of *Bcl*-*2* induced by BPA exposure (Figure 4D).

### 2.5. The Effects of Na_2_SeO_3_ and SeNPs on Inflammatory Pathways in BPA-exposed IPECs

**Figure 5:**
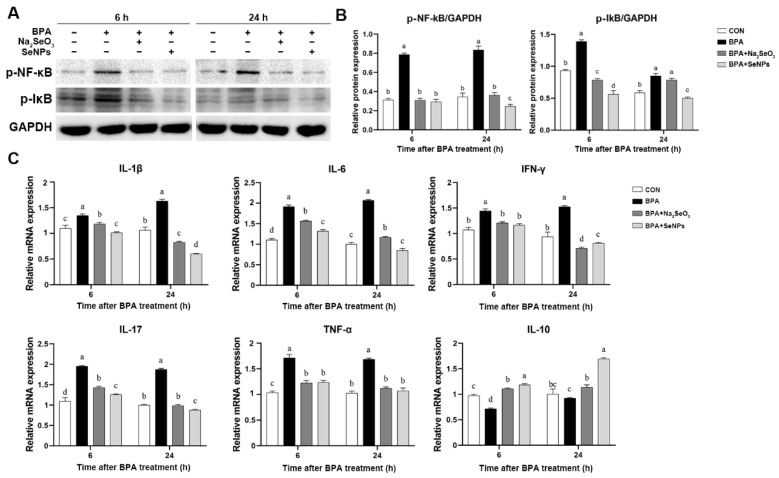
Effect of Na_2_SeO_3_ and SeNPs treatment on the inflammatory response of IPEC-J2 cells after BPA exposure. Representative panels showing expression of p-NF-κB and p-IκB by Western blotting at 6 and 24 h after BPA exposure (**A**). Expression of GAPDH was measured as an internal control. Results are presented as the ratio of p-NF-κB and p-IκB band intensity to that of GAPDH (**B**). The relative expression of mRNAs for the *IL-1β, IL-6, IFN-γ, IL-17, TNF-α*, and *IL-10* genes was analyzed by quantitative real-time PCR (**C**). Data are presented as the means ± SEM of three independent experiments. ^a,b,c,d^ Mean values within a row with different superscript letters were significantly different (*p* < 0.05).

The Western blot results showed that cells exposed to BPA alone had a higher expression of p-NF-κB and p-IκB proteins compared with the untreated control cells at 6 and 24 h, but this increase was inhibited by SeNPs or Na_2_SeO_3_ treatment (Figure 5A,B). SeNPs exhibited a better inhibitory effect on the BPA-induced increase in the p-NF-κB expression at 24 h and in the p-IκB expression at 6 and 24 h. Compared with the untreated control group, BPA exposure increased the mRNA expression of pro-inflammatory cytokine *IL-1β*, *IL-6*, *IFN-γ*, *IL-17*, and *TNF-α* at 6 and 24 h, but this increase was attenuated by SeNPs or Na_2_SeO_3_ treatment (Figure 5C). Compared with cells in the Na_2_SeO_3_ + BPA group, cells in the SeNPs + BPA group had a lower expression of *IL-1β*, *IL-6*, and *IL-17* during BPA exposure. Compared with the untreated control group, BPA exposure led to decreased mRNA expression of *IL-10*, but this decrease was reversed by SeNPs or Na_2_SeO_3_ treatment.

### 2.6. SeNPs Maintained the Antioxidant Capacity of IPEC-J2 Cells during BPA Exposure

**Figure 6:**
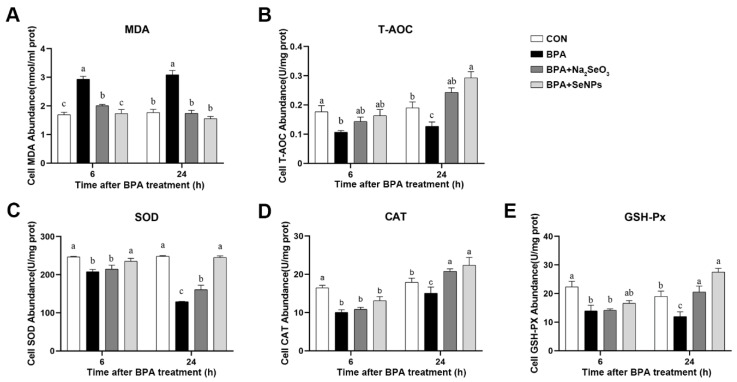
Effects of Na_2_SeO_3_ and SeNPs treatment on oxidative stress of IPEC-J2 cells after BPA exposure. Oxidative stress was analyzed by measuring the level of MDA (**A**), T-AOC (**B**), SOD (**C**), CAT (**D**), and GSH-Px (**E**). Data are presented as the means ± SEM of three independent experiments. ^a,b,c,^ Mean values within a row with different superscript letters were significantly different (*p* < 0.05).

The antioxidant capacity of IPEC-J2 cells was assessed by measuring the abundance of malondialdehyde (MDA), total antioxidant capacity (T-AOC), superoxide dismutase (SOD), catalase (CAT), and glutathione peroxidase (GSH-Px). Compared with the untreated control group, BPA exposure increased the abundance of MDA at 6 and 24 h, and this increase was inhibited by SeNPs or Na_2_SeO_3_ treatment (Figure 6A). Compared with the untreated control group, BPA resulted in decreased abundance of T-AOC, SOD, CAT, and GSH-Px at 6 and 24 h (Figure 6B–E). SeNPs or Na_2_SeO_3_ treatment inhibited even reversed the BPA-induced decrease in the abundance of T-AOC, SOD, CAT, and GSH-Px at 24 h compared with BPA alone group. SeNPs, but not Na_2_SeO_3_ treatment, reversed the decreased abundance of SOD induced by BPA at 6 h.

### 2.7. SeNPs Ameliorated the BPA-Induced ERS in IPEC-J2 Cells

**Figure 7:**
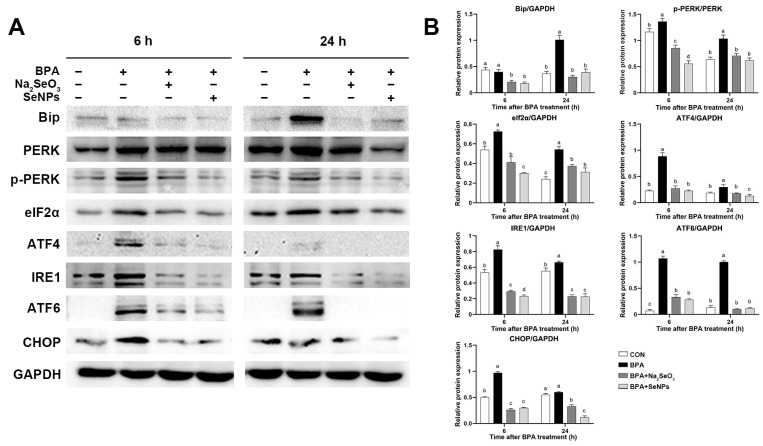
Effects of Na_2_SeO_3_ and SeNPs treatment on ER stress of IPEC-J2 cells after BPA exposure. Representative panels showing expression of Bip, PERK, p-PERK, ATF4, ATF6, eIf2α, p-IRE1, and CHOP proteins by Western blotting (**A**). Expression of GAPDH was measured as an internal control. The cellular protein PERK was used as an internal control for p-PERK, and GAPDH was used as an internal control for other proteins (**B**). Data are presented as the means ± SEM of three independent experiments. ^a,b,c,d^ Mean values within a row with different superscript letters were significantly different (*p* < 0.05).

Compared with untreated control cells, BPA exposure led to increased expression of ERS marker protein Bip at 24 h, whereas this increase was attenuated by SeNPs or Na_2_SeO_3_ treatment (Figure 7A,B). For the PERK/ATF4 pathway, BPA exposure increased the expression of p-PERK, translational initiation factor 2α (eIf2α), and ATF4 proteins at 6 and 24 h, and this increase was inhibited by SeNPs or Na_2_SeO_3_ treatment. Compared with Na_2_SeO_3_ treatment, cells treated with SeNPs showed lower expression of p-PERK and eIf2α at 6 h and lower expression of ATF4 at 24 h (Figure 7A,B). For the IRE1 and ATF6 pathways, BPA exposure increased the expression of IRE1 and ATF6 compared with the untreated control group, and this increase was inhibited by SeNPs or Na_2_SeO_3_ treatment. SeNPs treatment exhibited a better inhibitory effect on the increased IRE1 protein expression induced by BPA at 6 h than Na_2_SeO_3_ treatment (Figure 7A,B). Compared with untreated control cells, BPA exposure led to increased expression of C/EBP homologous protein (CHOP) at 6 h, whereas this increase was attenuated by SeNPs or Na_2_SeO_3_ treatment.

## 3. Discussion

As a common environmental contaminant, BPA has been shown to cause potential damage to many tissues and organs (lung, liver, kidney, skin, and mucous membrane) of humans and animals [30,31]. The intestine is especially vulnerable to the adverse effects of BPA. Previous studies showed that selenium and its nano form have powerful protective effects against oxidative stress, DNA damage, and apoptosis in response to BPA cisplatin and ionizing radiation exposure in vivo [32,33]. In this study, we employed the IPEC-J2 cell line, widely used in mimicking intestinal epithelial cells in in vitro studies, to evaluate the adverse effects of BPA exposure on the intestinal epithelial barrier function and further revealed the protective effects and mechanism of SeNPs. Our results showed that SeNPs alleviated the development of BPA-induced toxicity and maintained the intestinal barrier functions by inhibiting ER stress and downstream intestinal epithelial cell apoptosis, inflammation, and oxidative stress.

The intestinal epithelial barrier acts as the first line of defense and plays a vital role in nutrition and immunoregulation. A layer of epithelial cells bounds together via intercellular junction proteins and maintains intestinal barrier integrity. Dietary BPA uptake destroys the morphology of the colonic epithelium and increases the pathology score by decreasing the expression of tight junction proteins (ZO-1, occludin, and claudin-1) in the colonic epithelium of mice [34]. The destruction of the intestinal barrier helps BPA to penetrate the intestine and then invade other organ systems. Dietary exposure of 50 μg/kg/day of BPA induces increased intestinal permeability and consequently leads to hepatic steatosis in CD-1 mice [35]. Studies found that long-term exposure to BPA for 22 weeks reduced the tight junctions in the colon of male C57BL/6J mice, resulting in the dysfunction of the gut barrier and impaired cognitive function [36]. Consistent with previous studies, our results showed that BPA exposure significantly decreased the expression of tight junction proteins both at 6 and 24 h. However, SeNPs treatment maintained the normal expression of tight junction proteins during BPA exposure at 24 h. We found that the protective effects of SeNPs were superior to Na_2_SeO_3_, as shown that Na_2_SeO_3_ treatment led to a lower expression of ZO-1 and Occludin proteins than BPA exposure alone. In a model of fluorine-induced chronic oxidative stress of broilers, selenomethionine exhibited a better protective effect on ameliorating tight junction network impairment than sodium selenite [37]. Nano-bio selenium can effectively improve the performance and intestinal integrity of broilers compared to the common organic and inorganic sources of selenium [38]. Our results suggest that SeNPs maintained the intestinal barrier function by increasing the expression of tight junction proteins to attenuate the adverse effects of BPA.

Apoptosis is programmed death of a self-protective nature after the host cell senses external risk factors [39]. There are two main pathways of apoptosis, the extrinsic apoptotic pathway mediated by caspase 8 and the endogenous pathway mediated by Bcl 2, caspase 3, caspase 7, and caspase 9 [40]. Studies have shown that cell apoptosis is the main signal pathway of BPA-induced tissue damage, including the brain [41], liver [42], and lung [43]. It has been shown that BPA-treated rat and human stem cells have a significant decrease in cell viability and substantial apoptosis as early as 10 min of exposure and at physiologically relevant low doses [44]. At the same time, it was found that BPA induced apoptosis of mouse interstitial cells through oxidative stress [45]. Treatment with Se significantly alleviated Cd-induced apoptosis in chicken livers, as evidenced by a reduction in the production of NO, iNOS activity, the number of apoptotic cells, and mRNA and protein expression levels of caspase-3 and Bax [46]. In the rat thyroid follicular cell model, selenium reduced the proportion of cell death. In addition, high doses of nano-selenium incubation may prevent tunicamycin-induced ER stress cell apoptosis through the maintenance of membrane integrity and the reduction of caspase 3/7 activity [47]. In the present study, BPA exposure decreased the expression of anti-apoptotic Bcl-2 and Bcl-xl, while conversely, BPA increased the Bax/Bcl-2 ratio and the expressions of pro-apoptotic Bax and effector protein caspase 3, caspase 8, and caspase 9, indicating that BPA had a strong pro-apoptotic effect on pig intestinal epithelial cells. A larger ratio of Bax/Bcl-2 directs cells toward apoptosis [48]. SeNPs or Na_2_SeO_3_ treatment alleviated the above apoptosis marker protein during BPA exposure at 6 and 24 h. Compared with the Na_2_SeO_3_ treatment, SeNPs treatment exhibited a better inhibitory effect on BPA-induced intestinal epithelial cell apoptosis, as shown by the lower expression of caspase-9 and Bax and the higher expression of Bcl-2 in the SeNPs group at 6 and 24 h.

The transcription factor NF-κB, as an important immunoregulatory factor, promotes the high expression of pro-inflammatory cytokine TNF-α and interleukin family, which have a significant disruptive effect on the expression and distribution of tight junction proteins and impair the intestinal epithelial barrier function [49,50]. BPA has been demonstrated to modulate the function of the immune system and increases the susceptibility to infections by virtue of acting as a pro-inflammatory molecule [51]. Long-term exposure to BPA-induced TLR4-dependent hypothalamic inflammation exacerbates diet-induced prediabetes [52]. Oral exposure to 4 µg/kg bw/d BPA exacerbates allergic inflammation in a mouse model of food allergy [53]. Consistent with previous reports, the current study found that BPA promoted the activation of NF-κB and inhibitor of NF-κB (IκB) and significantly elevated the expression of intestinal inflammation-related genes, including IL-6, IL-17, IL-1β, IFN-γ, and TNF-α. These results reflected that the activation of innate immune responses was an important mechanism of BPA-induced impairment of the intestinal epithelial barrier function. Selenium can control exaggerated immunological responses and persistent inflammation [54]. In this study, compared with Na_2_SeO_3_, SeNPs could reduce the excessive expression of inflammatory factors caused by BPA, indicating selenium nanoparticles exhibit considerable promise as a more effective and reliable tool in controlling and preventing intestinal inflammation during BPA exposure.

Oxidative stress refers to an imbalance between oxidants and antioxidants in the body produced by free radicals and is known to be a significant factor influencing disease occurrence [55]. BPA can increase MDA content in rat testicular tissue and thus leads to oxidative stress and functional damage [56]. Selenium modulates antioxidant system status by affecting the activity of antioxidant enzymes via selenoproteins and selenic nucleic acids [57,58]. Dietary intake of organic selenium can increase the activity of GSH-Px and significantly decrease the content of MDA in the serum and placenta of the sows [59]. In this study, BPA exposure led to a decrease in the content of T-AOC, SOD, CAT, and GSH-Px and an increase in the content of MDA, while SeNPs or Na_2_SeO_3_ treatment alleviated the effects of BPA on oxidative stress. We also found that cells pretreated with SeNPs had a higher abundance of T-AOC, SOD, CAT, and GSH-Px and a lower content of MDA compared with cells pretreated with Na_2_SeO_3_. These results were in line with the study reporting that dietary supplement of nano-Se exhibits better effects on elevating the levels of GSH-Px and T-AOC in laying hens compared with Na_2_SeO_3_ [60].

Under the stimulation of the endoplasmic reticulum by intracellular and extracellular stress factors, the number of unfolded and misfolded proteins in ER increases abnormally, which induces ER stress. After ER stress occurs, UPR is activated to clear unfolded and misfolded proteins. ER stress is considered an intermediate pathway to induce the other pathways and damage cells during BPA exposure. Single or combined exposure to BPA and mono(2-ethylhexyl) phthalate cause serious toxicological outcomes in the HepG2 cell line, including altered oxidant/antioxidant status, aggravated ER stress, and apoptosis [61]. BPA exposure induces neurotoxicity in rats involved with neuronal ER stress, apoptosis, and JAK1/STAT1 signaling pathway [41]. Long-term BPA exposure disturbed lipid metabolism and induced oxidative stress, ER stress, apoptosis, autophagy, and inflammatory response in the liver of common carp [42]. BPA triggers apoptosis by activating ER stress in human endometrial stromal cells [62]. ER stress might be related to the disturbance of the intestinal barrier in IPEC-J2 cells after heat exposure [63]. Pterostilbene restores the intestinal barrier function by inhibiting ER stress in piglets [64]. It was reported that selenium deficiency leads to apoptosis by inducing ER stress in the swine small intestine and IPEC-J2 cells, indicating that selenium has a protective effect on porcine intestinal health through modulating ER stress [65]. A previous study showed that ER stress and oxidative stress showed a high correlation, and the lack of selenium triggers ER stress and oxidative stress, and apoptosis in chicken hepatocytes [66]. In this study, we determined the status of three UPR pathways mediated by receptor proteins PERK, IRE1, and ATF6. BPA exposure significantly activated UPR, as shown by the increased expression of Bip, PERK, eIf2α, ATF4, IRE1α, ATF6, and CHOP proteins. Interestingly, we also found that the activated UPR was mitigated by SeNPs and Na_2_SeO_3_ treatment. Here, BPA exposure disrupted tight junction function and induced proinflammatory response, oxidative stress, apoptosis, and ER stress in intestinal epithelial cells, while SeNPs treatment could alleviate these effects.

In conclusion, our results suggested that, compared with inorganic selenium, selenium nanoparticles were superior in alleviating BPA-induced proinflammatory response, oxidative stress, and apoptosis in porcine intestinal epithelial cells through ameliorating ER stress, thus maintaining the intestinal epithelial barrier function. Our study provides a theoretical basis for the toxic mechanism of BPA in swine and further complements the risk assessment of BPA in domestic animals, and pushes forward a solution for BPA-induced damage by probing the role of selenium nanoparticles.

## 4. Materials and Methods

### 4.1. Preparation of SeNPs

The chitosan was dissolved in deionized water and stirred for 6 h. The chitosan (2 mg/mL) was mixed vigorously with the freshly prepared sodium selenite solution (20 mmol/L) at a ratio of 1:4 (*v/v*) for 60 min. Then, the Vc solution (80 mmol/L) was added to the sodium selenite solution with an equal volume, stirred at 35 °C for 12 h, and lyophilized to obtain SeNPs.

### 4.2. Dynamic Light Scattering (DLS)

DLS was employed to record the variation in the intensity of the scattered light on the microsecond time scale based on particles in gas or liquid being subjected to Brownian motions. To investigate the stability of colloidal particles, the Z-average size and polymer dispersity index (PDI) of SeNPs were measured by the Nanosizer ZS90 instrument (Malvern Instruments, Malvern, UK). The refractive indices of water and the SeNPs solution were taken as 1.333. In general, zeta potentials reflect the electrostatic repulsion between dispersed particles, and a high value of zeta potentials represents a good dispersion of colloid particles. In this work, zeta potentials were performed by using Nanosizer. All measurements were conducted at 25 ± 2 °C in triplicate.

### 4.3. Transmission Electron Microscopy (TEM)

SeNPs were fixed in 2.5% glutaraldehyde for 4 h and then in 1% osmium tetroxide for 1.5 h at 4 °C, after which they were dehydrated in gradient ethanol solutions and propylene oxide. The resin was then impregnated with acetone for 12 h and polymerized on a polymerized plate at 40 °C for 48 h. Ultrathin sections (1 mm) were cut with the Ultramicrotome Leica EM UC7 (Leica, Wetzlar, Germany) and then stained with uranyl acetate and lead citrate [67]. The sections were examined under a TEM (JEM-2100, JEOL, Tokyo, Japan).

### 4.4. Gastric and Intestinal Fluid Digestion Test

For the gastric fluid digestion test, 1 mg of SeNPs was mixed with 10 mL of gastric fluid (pepsin, ≥30 U/mL) and adjusted to pH 1.2 with 6 M HCl. Then, the mixture was incubated at 37 °C for 3 h in a thermostat water bath. The samples were taken at the digestion time of 0, 1, 2, and 3 h. For the intestinal fluid digestion test, after the gastric digestion for 4 h was centrifuged at 10,000× *g* for 30 min, the precipitation was collected and mixed with 10 mL of intestinal fluid (pancreatin, ≥40 U/mL). The mixture was then adjusted to pH 7.4 with 1 mol/L of NaHCO_3_, followed by incubation at 37 °C for 3 h in the thermostat water bath. The samples were taken at the digestion time of 0, 1, 2, and 3 h.

### 4.5. Cell Culture and Treatment

The IPEC-J2 cell line is a porcine colonic epithelial cell line. Cryopreserved cells were extracted from liquid nitrogen and continuously cultured for three passages for subsequent experiments. The cells were cultured in DMEM-F12 containing 10% fetal bovine serum (Invitrogen, Carlsbad, CA, USA) at 37 °C in an atmosphere of 5% CO_2_ and 95% air at 95% relative humidity. The medium was replaced every 2 days. The cells were treated under 1 of 4 conditions, as follows: (i) medium (CON group); (ii) 50 μmol of BPA (RHAWN, China) exposure (BPA group); (iii) pre-incubation with 32.85 μg/mL of sodium selenite following 50 μmol of BPA exposure (BPA + Na_2_SeO_3_ group); (iv) pre-incubation with 15 μg/mL of SeNPs following 50 μmol of BPA exposure (BPA + SeNPs group). Cell samples were harvested at 6 and 24 h after BPA exposure, respectively.

### 4.6. Cell Counting Kit-8 (CCK8) Assay

IPEC-J2 cells were seeded at a density of 1 × 10^4^ per well in 96-well plates and cultured for 24 h. According to the BPA concentration range reported in recent research, cells were treated with 0, 10, 20, 40, 50, 60, and 80 μM of BPA for 6 and 24 h, respectively. The final DMSO concentration in all cell cultures was adjusted to 0.01% (*v/v*). A Cell Counting Kit-8 (Solarbio, Beijing, China) was employed to determine cell viability. The absorbances at 450 nm were determined by a microplate reader (INNO-M, TeCK, China). The highest concentration of BPA that did not considerably impact cell viability was chosen as the favorable concentration for the subsequent assays. All the experiments were carried out independently in triplicate.

### 4.7. Morphological Observation

Morphological Observation: The cells were seeded into a 6-well plate, and different BPA, Na_2_SeO_3_, or SeNPs treatments were carried out. At 24 h after BPA exposure, cell morphology was observed under a light microscope (Olympus Corporation, Tokyo, Japan).

### 4.8. Western Blotting

Total protein was extracted by lysis buffer for Western blotting with 100 mM of phenylmethanesulfonylfluoride, and 25 μg of total protein sample was subjected to SDS-polyacrylamide gel electrophoresis under reducing conditions. Separated proteins were transferred to nitrocellulose membranes in Tris-glycine buffer containing 20% methanol at 4 °C. The membranes were blocked with 5% skim milk for 2 h, following incubated overnight with diluted primary antibodies against Bcl-xl (1:5000, Abcam, Cambridge, UK), caspase 3 (1:2000, Cell signaling technology, Boston, MA, USA), Bip (1:2000, Cell signaling technology), PERK (1:5000, Abcam), p-PERK (1:1000, Bioss), ATF4 (1:2000, Proteintech, Chicago, IL, USA), ATF6 (1:2000, Proteintech), eIf2α (1:1000, Proteintech, Wuhan, China), p-IRE1(1:1000, Bioss), CHOP (1:1000, Bioss), NF-κB (1:1000, Bioss), IκB (1:2000, Cell signaling technology), ZO-1 (1:1000, Bioss, Beijing, China), Occludin (1:1000, Bioss), Claudin-1 (1:1000, Bioss), and GAPDH (1:1000, Proteintech, China) followed by goat anti-rabbit IgG (H+L; 1:1000, Proteintech, China). The gray values of protein bands were measured by ImageJ software version 6.1 (Bio-Rad Laboratories, Hercules, CA, USA). The cellular protein PERK was used as an internal control for p-PERK, and GAPDH was used as an internal control for other proteins.

### 4.9. Quantitative Real-Time PCR (qRT-PCR) Analysis

Total RNA samples were isolated by Trizol reagent (Invitrogen) according to the manufacturer’s instructions. The RNA was reverse transcribed by a cDNA synthesis kit (Promega, Madison, WI, USA). Specific primers (*caspase-8*, *caspase-9*, *Bcl-2*, *Bax*, *Il-1β*, *Il-6*, *Il-17*, *Il-10*, *Tnf-*, *Ifn-γ*, and *Gapdh*) were designed by Primer-BLAST at the National Center for Biotechnology Information and were shown in Table 1. The dried RNA pellets were resuspended in 50 μL of diethylpyrocarbonate-treated water. The concentration and purity of the total RNA were determined using a spectrophotometer. cDNA was synthesized from 1 μg of the total RNA using oligo dT primers and Superscript II reverse transcriptase according to the manufacturer’s instructions (Promega, Beijing, China), and cDNA was stored at 80 °C. Reactions were performed in a 20 μL of reaction mixture containing 10 μL of 2X SYBR Green I PCR Master Mix,1 μL of cDNA, 1 μL of each primer (10 μM), and 7 μL of PCR-grade water. The optimal conditions for PCR were 95 °C for 2 min, followed by 40 cycles of denaturation for 15 s at 95 °C, annealing for 1 min at 60 °C, and elongation for 50 s at 72 °C. The qRT-PCR was performed with StepOne^TM^ 96 system (Roche, Basel, Switzerland). The relative abundance of mRNA of each gene was calculated according to the 2^−∆∆Ct^ metho d and was normalized to the mean expression of GAPDH.

### 4.10. Antioxidant Determination

The activities of antioxidant GSH-PX (A005-1-1), catalase (CAT, A007-1-1), superoxide dismutase (SOD, A001-3-1), total antioxidant capacity (T-AOC, A015-2-1), and the concentration of malondialdehyde (MDA, A003-1-1) were analyzed using the corresponding commercial assay kit (Nanjing Institute of Jiancheng Biological Engineering, Nanjing, China) according to the manufacturer’s instructions. The antioxidant activity of the above parameters was calculated based on the protein content of the cell samples, and the assay was performed using the method described by Bradford.

### 4.11. Detection of Apoptosis by Flow Cytometry

The percentage of apoptotic cells was determined using a commercial Annexin V-FITC/PI Apoptosis Detection Kit (Kangwei Biotech, Shenzhen, China). Following the manufacturer’s instructions, the harvested cell pellets were washed twice with pre-cooled PBS and then resuspended in 0.5 mL of 1X binding buffer. Afterward, cells were incubated with FITC-labeled Annexin V for 15 min and were stained with 50 μg/mL of propidium iodide for 5 min before detection. Samples were analyzed by a flow cytometer (Beckman, Brea, CA, USA). The data analysis was performed using CytExpert software version 2.4 (Beckman Coulter, Brea, CA, USA), and the apoptosis percentage was referred to as the ratio of apoptosis cells to total cells.

### 4.12. Statistical Analysis

All of the experimental data are expressed as the means ± SEM. The differences between the groups were determined by a 1-way analysis of variance (ANOVA) test followed by Tukey’s honestly significant difference post hoc test using GraphPad Prism 5 software (Graphpad Software Inc., San Diego, CA, USA). A *p*-value of <0.05 was considered indicative of statistical significance.

## Figures and Tables

**Table 1:** Sequences of oligonucleotide primers used for real-time PCR, length of the respective PCR product, and gene accession number.

Gene Product ^a^	Primer Direction ^b^	Sequence (5′ to 3′)	Product Size (bp)	Accession Number
*GADPH*	F	CCAGAACATCATCCCTGCTT	229	XM_021091114
	R	GTCCTCAGTGTAGCCCAGGA		
*Caspase-8*	F	TGGAGGACGTTTTCACAGGGC	133	XM_021074712
	R	AGTTGTAACCGGAGGCAAATCC		
*Caspase-9*	F R	AACTTCTGCCATGAGTCGGG GAGGTGGCTGGCCTTGG	135	XM_013998997
*Bcl-2*	F	AGCATGCGGCCTCTATTTGAT	107	XM_021099593
	R	CACTTATGGCCCAGATAGGCA		
*Bax*	F	AGCAGATCATGAAGACAGGGG	137	XM_003127290
	R	ACACTCGCTCAACTTCTTGGT		
*IL-6*	F	GGCTGTGCAGATTAGTACC	124	JQ_839263
	R	CTGTGACTGCAGCTTATCC		
*IL-10*	F	CTTGTTGCTGACCGGGTCTC	110	HQ_236499
	R	TCTCTGCCTTCGGCATTACG		
*IL-17*	F	GACGGCCCTCAGATTACTCC	125	KF_646141
	R	AGCATTGATACAGCCCGAGT		
*IL-1β*	F	GCCAACGTGCAGTCTATGGAGTG	91	XM_021085847
	R	GGTGGAGAGCCTTCAGCATGTG		
*TNF-α*	F	GCCCTTCCACCAACGTTTTC	97	JF_831365
	R	CAAGGGCTCTTGATGGCAGA		
*Ifn-γ*	F	CAGGCCATTCAAAGGAGCAT	150	MH_538101
	R	GAGTTCACTGATGGCTTTGCG		

^a^ GAPDH = glyceraldehyde-3-phosphate dehydrogenase. ^b^ F = forward; R = reverse.

## Data Availability

All data are presented within the manuscript.
